# Maternal Low-Protein Diet Affects Epigenetic Regulation of Hepatic Mitochondrial DNA Transcription in a Sex-Specific Manner in Newborn Piglets Associated with GR Binding to Its Promoter

**DOI:** 10.1371/journal.pone.0063855

**Published:** 2013-05-14

**Authors:** Yimin Jia, Runsheng Li, Rihua Cong, Xiaojing Yang, Qinwei Sun, Nahid Parvizi, Ruqian Zhao

**Affiliations:** 1 Key Laboratory of Animal Physiology and Biochemistry, Ministry of Agriculture, Nanjing Agricultural University, Nanjing, Jiangsu, China; 2 College of Veterinary Medicine, Northwest A & F University, Yangling, Shannxi, China; 3 Department of Functional Genomics and Bioregulation, Institute of Animal Genetics, FLI, Mariensee, Neustadt, Germany; University of Sydney, United States of America

## Abstract

Mitochondrial oxidative phosphorylation (OXPHOS) plays an important role in energy homeostasis by controlling electron transfer and ATP generation. Maternal malnutrition during pregnancy affects mitochondrial (mt) DNA-encoded OXPHOS activity in offspring, yet it is unknown whether epigenetic mechanism is involved in the transcriptional regulation of mtDNA-encoded OXPHOS genes. In this study, 14 primiparous purebred Meishan sows were fed either standard- (SP, 12% crude protein) or low-protein (LP; 6% crude protein) diets throughout gestation, and the hepatic expression and transcriptional regulation of mtDNA-encoded OXPHOS genes were analyzed in newborn piglets. Maternal low protein diet decreased hepatic mtDNA copy number in males, but not in females. LP male piglets had significantly higher hepatic AMP concentration and low energy charge, which was accompanied by enhanced mRNA expression of NADH dehydrogenase subunits 6, cytochrome *c* oxidase subunit 1, 2, 3 and cytochrome *b*, as well as increased cytochrome *c* oxidase enzyme activity. In contrast, LP female piglets showed significantly lower hepatic AMP concentrations and higher energy charge with no alterations in OXPHOS gene expression. Moreover, LP males demonstrated higher glucocorticoid receptor (GR) binding to the mtDNA promoter compared with SP males, which was accompanied by lower cytosine methylation and hydroxymethylation on mtDNA promoter. Interestingly, opposite changes were seen in females, which showed diminished GR binding and enriched cytosine methylation and hydroxymethylation on mtDNA promoter. These results suggest that maternal low protein diet during pregnancy causes sex-dependent epigenetic alterations in mtDNA-encoded OXPHOS gene expression, possibly GR is involved in mtDNA transcription regulation.

## Introduction

Mitochondria play an important role in maintaining energy homeostasis by balancing ATP generation and expenditure at the cellular level. Most of the ATP required to maintain the normal functions of the cell is produced through oxidative phosphorylation (OXPHOS) in mitochondria. Mitochondrial dysfunction in various tissues, such as skeletal muscle [1], liver [2] and fat [3], has been associated with metabolic diseases in human and animal models. Normally, it is considered that decreased mitochondrial OXPHOS activity is related to metabolic perturbation in diabetes human [4] and mice [5]. Recently, enhanced mitochondrial OXPHOS activity has been found to be associated with diabetes and obesity in mice [6], whereas impaired mitochondrial OXPHOS activity protects the mice against diabetes and obesity [7].

Growing evidence demonstrates that low birth weight due to maternal malnutrition is associated with an increased risk of metabolic syndrome in adult offspring [8], [9]. Deregulation of energy homeostasis is a common feature of metabolic syndrome and a number of studies have shown that maternal nutrition programs offspring mitochondria OXPHOS activity in various types of tissues, including liver [10], pancreatic islet [11] and skeletal muscle in rats [12], kidney [13] and skeletal muscle [14] in sheep, as well as liver in pig [15].

Liver plays a central role in energy homeostasis of the whole body and hepatic mitochondrial OXPHOS activity is potentially related to insulin resistence [16]. In mammals, 13 OXPHOS-related proteins are encoded by mitochondria's own genome (mtDNA) and their expression is under the control of various hormones. Triiodothyronine (T_3_) and glucocorticoid are reported to increase mitochondrial gene expression and OXPHOS activity [17], [18] through binding to their receptors, respectively. Previous studies indicate that T_3_ receptor and glucocorticoid receptor (GR) could be imported into mitochondria, and T_3_ response elements (TREs) and glucocorticoid response elements (GREs) have been identified in the control region of mtDNA, i.e., the promoter of mtDNA-encoded genes [19], [20]. However, it remains to be elucidated whether and how maternal nutrition affects transcription of mtDNA-encoded OXPHOS genes in the liver of offspring.

It has been well documented that prenatal malnutrition may cause long-term changes of gene expression through altering DNA methylation on the promoter of nuclear genes, such as glucocorticoid receptor in rat [21], phosphoenolpyruvate carboxykinase 1 in baboon [22], and glucose-6-phosphatase in pig [23]. DNA methylation is particularly vulnerable during the very early stage of mammalian development, which is a crucial period for de novo DNA methylation and reprogramming of DNA methylation patterns [24]. In addition, 5-methylcytosine (5mC) and 5-hydroxymethylcytosine (5hmC) are reported to present in mammalian mtDNA, and DNA methyltransferase 1 (DNMT1) is found to translocate to the mitochondria to regulate cytosine methylation and hydroxymethylation in mammals [25]. Nevertheless, it is unclear whether maternal malnutrition may program hepatic mtDNA cytosine methylation and hydroxymethylation in offspring.

Therefore, the present study was aimed to answer two questions: first, to assess the impact of maternal dietary protein on hepatic expression of mtDNA-encoded OXPHOS genes in neonatal piglets; and second, to determine whether such effects involve mtDNA transcriptional regulation, including GR binding, DNA methylation and hydroxymethylation on the control region of mtDNA in the liver of newborn piglets.

## Results

### The characteristics of newborn piglets

Maternal low protein diet throughout gestation significantly decreased body weight (*P*<0.01) and liver weight (*P*<0.01) in newborn piglets, there is diet and sex interaction in BMI of newborn piglets, the BMI (*P*<0.05) was significantly decreased in LP female offsprings compared to LP male offsprings. While maternal low protein did not affect serum concentrations of T3, T4 or cortisol. However, significant sex effect was observed for body weight and serum cortisol concentration, males showing higher body weight (*P*<0.05) as well as the serum cortisol level (*P* = 0.07) compared to females (Table 1).

**Table 1:** Effect of maternal LP diet on body weight, liver weight and hormones in newborn piglets.

Parameter	Male		Female		2-way ANOVA p-value		
	SP	LP	SP	LP	D	S	D × S
BW *kg*	0.97±0.03	0.85±0.03	0.92±0.02	0.81±0.03	0.05	0.04	0.88
BMI g/m^2^	20.2±1.60ab	20.4±1.12ab	24.1±1.72a	18.9±1.08b	0.06	0.36	0.05
LW	26.5±1.90	21.4±0.90	26.6±1.90	20.5±0.70	<0.01	0.66	0.81
LW/BW *g/kg*	27.2±1.70	24.9±1.30	28.1±1.90	24.9±1.30	0.09	0.77	0.78
Cortisol *mg/L*	0.35±0.05	0.33±0.03	0.29±0.03	0.25±0.03	0.58	0.07	0.99
T3 *µg/L*	0.98±0.35	1.03±0.19	0.87±0.08	0.85±0.11	0.97	0.48	0.86
T4 *µg/L*	84.9±6.76	85.6±6.69	78.1±9.58	82.7±4.48	0.71	0.49	0.78
T4/T3	127±26.7	99.3±13.1	91.5±10.3	106±10.7	0.69	0.38	0.20

Values are mean ± SEM, Means without a common letter differ, *P*<0.05. BW, body weight; D, diet; LP, low protein; LW: liver weight; S, sex; SP, standard protein; T3, Triiodothyronine; T4, thyronine.

### The content of ATP, ADP, AMP, NAD, NADH and mtDNA copy number in liver

**Figure 1:**
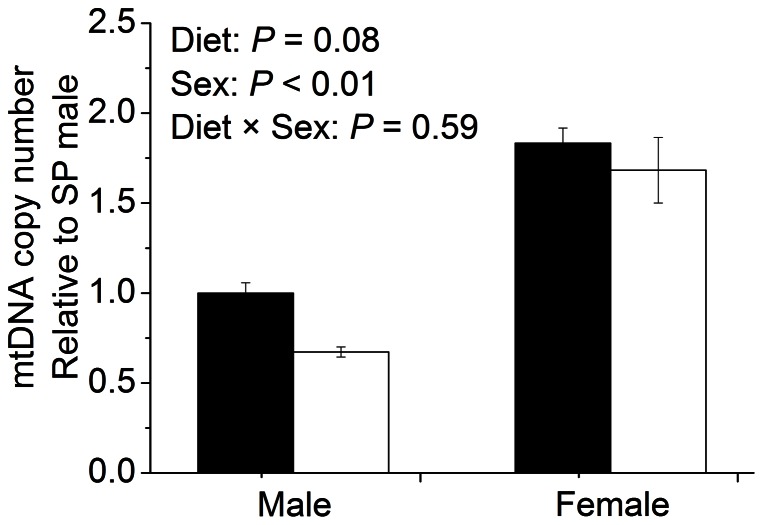
The mtDNA copy number in liver. Values are mean ± SEM, Means without a common letter differ, *P*<0.05. Filled bar, standard protein diet; blank bar, low-protein diet. mtDNA, mitochondrial DNA.

Hepatic NADH concentration (*P*<0.01), as well as the ratio of NADH to NAD (*P*<0.01), were significantly higher in LP piglets compared with SP piglets (Table 2). Significant diet and sex interactions were observed for the hepatic AMP concentration and the energy charge, suggesting sex disparities in the adaptation responses to maternal low protein diet (Table 2). Male piglets in LP group demonstrated significantly higher hepatic AMP concentration (*P*<0.05) and low energy charge (EC), whereas the opposite was true for the females showing significantly lower hepatic AMP concentrations (*P*<0.05) and higher EC (*P*<0.01) in LP group. Moreover, a significant sex difference was observed for mtDNA copy number, females showed significantly (*P*<0.01) more mtDNA content compared to males (Figure 1). Maternal low protein diet trended to decrease hepatic mtDNA copy number (*P* = 0.08), but only males showed significant reduction in mtDNA copy number (*P*<0.05).

**Table 2:** Effect of maternal LP diet on hepatic ATP, ADP, AMP, NADH and NAD in newborn piglets.

Parameter	Male		Female		2-way ANOVA p-value		
	SP	LP	SP	LP	D	S	D × S
ATP *µmol/g, liver*	1.48±0.12	1.55±0.11	1.22±0.09	1.69±0.22	0.06	0.64	0.17
ADP *µmol/g, liver*	1.18±0.16	1.42±0.21	1.12±0.18	1.32±0.14	0.25	0.69	0.93
AMP *µmol/g, liver*	4.91±0.59ab	6.12±0.26a	5.99±0.75a	3.32±0.81b	0.31	0.23	0.01
EC	0.36±0.01ab	0.32±0.01b	0.29±0.03b	0.47±0.08a	0.76	0.28	0.01
NADH *µmol/g, liver*	0.40±0.09	0.69±0.06	0.36±0.10	0.65±0.04	0.01	0.66	0.97
NAD *µmol/g, liver*	3.45±0.26	3.73±0.24	3.87±0.26	3.38±0.18	0.66	0.90	0.14
NADH/NAD	0.10±0.02	0.16±0.01	0.08±0.02	0.17±0.01	0.01	0.83	0.50

Values are mean ± SEM, Means without a common letter differ, *P*<0.05. D, diet; EC, energy charge; LP, low protein; S, sex; SP, standard protein. EC was calculated using the following formula:

EC  =  ([ATP] +1/2[ADP])/([ATP] + [ADP] + [AMP]).

### Mitochondrial DNA-encoded mRNA expression and cytochrome *c* oxidase (COX) enzyme activity

**Figure 2:**
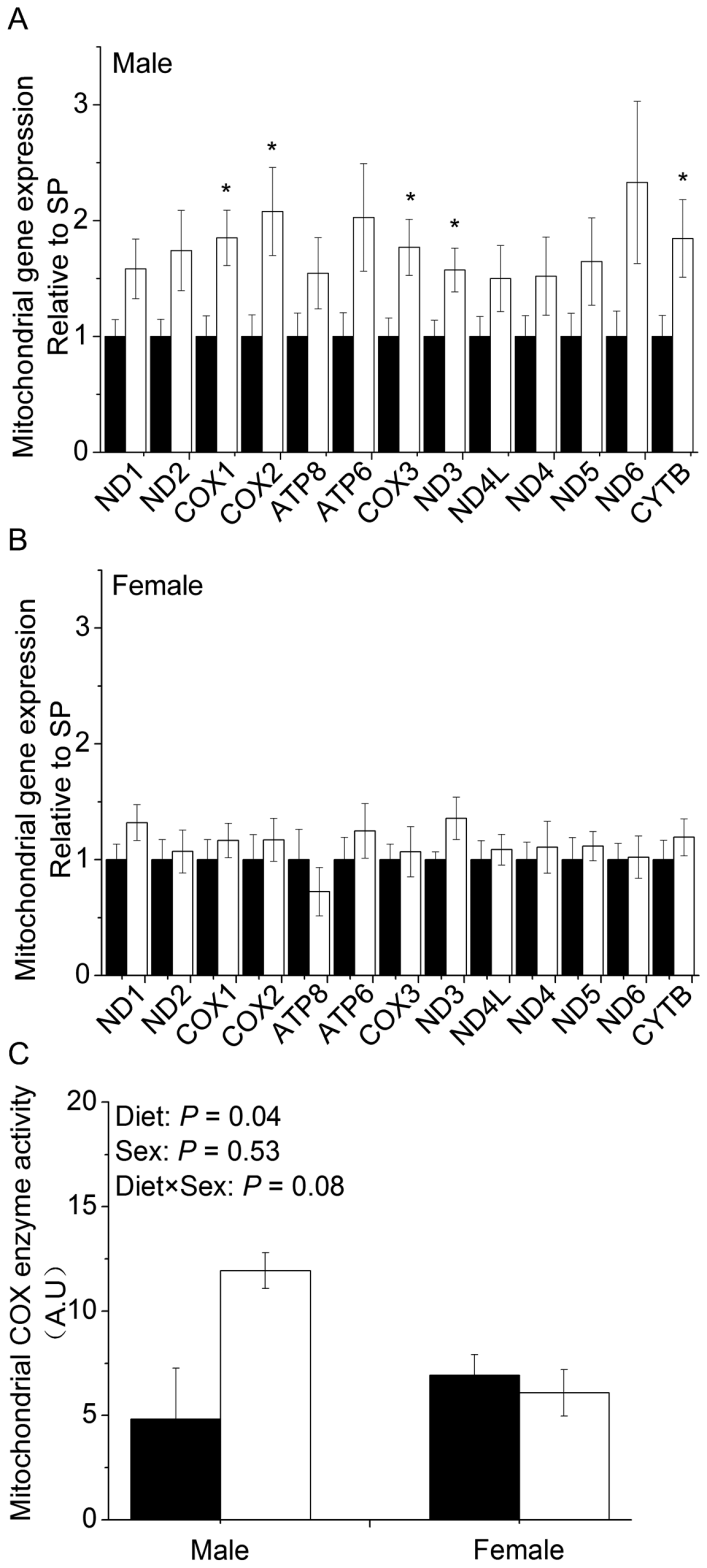
Expression of mtDNA-encoded genes and COX enzyme activity in liver. (A) Expression of mtDNA-encoded genes in liver of male newborn piglets. (B) Expression of mtDNA-encoded genes in liver of female newborn piglets. (C) COX enzyme activity in liver of both male and female newborn piglets. Filled bar, standard protein diet; blank bar, low-protein diet. Values are mean ± SEM, * *P*<0.05 vs. SP of the same sex. Means without a common letter differ, *P*<0.05.

The mRNA abundances of 13 genes involved in hepatic OXPHOS were determined by real-time PCR, and a sexual dimorphic response of these genes to maternal LP diet was observed in the liver of newborn piglets. Among these genes, *COX1* (*P*<0.05), *COX2* (*P*<0.05), *COX3* (*P*<0.05), *NADH dehydrogenase subunit 3* (*ND3*) (*P*<0.05) and *cytochrome b* (*CYTB*) (*P*<0.05) were significantly up-regulated in the liver of LP males compared to SP males (Figure 2A), but no difference was observed in females (Figure 2B). In agreement with mRNA abundances, mitochondrial COX enzyme activity (Figure 2C) was significantly enhanced (*P*<0.05) in male piglets born to sows fed LP diet.

### Hepatic GR expression and GR binding to the control region of mtDNA

**Figure 3:**
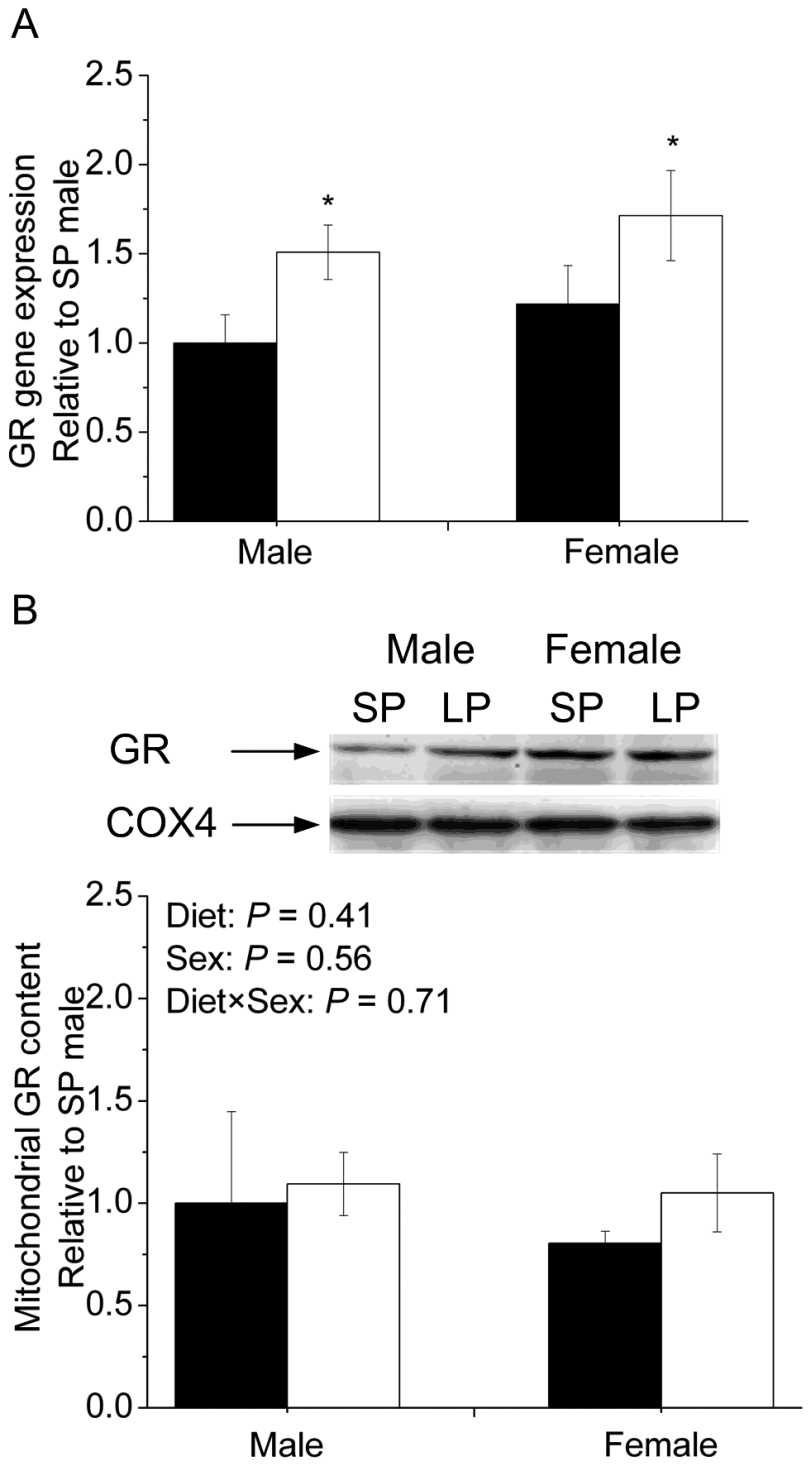
The levels of GR mRNA and protein in liver. (A) Hepatic GR gene expression in both male and female newborn piglets. (B) The content of hepatic GR protein in both male and female newborn piglets. Filled bar, standard protein diet; blank bar, low-protein diet. Values are mean ± SEM, * *P*<0.05 vs. SP of the same sex.

*GR* mRNA expression was significantly up-regulated (*P*<0.05) in the liver of both male and female LP piglets (Figure 3A). Although mitochondria content of GR protein in the liver of newborn piglets was not affected, GR binding to the control region of mtDNA was altered by both diet and sex (Figure 3B).

**Figure 4:**
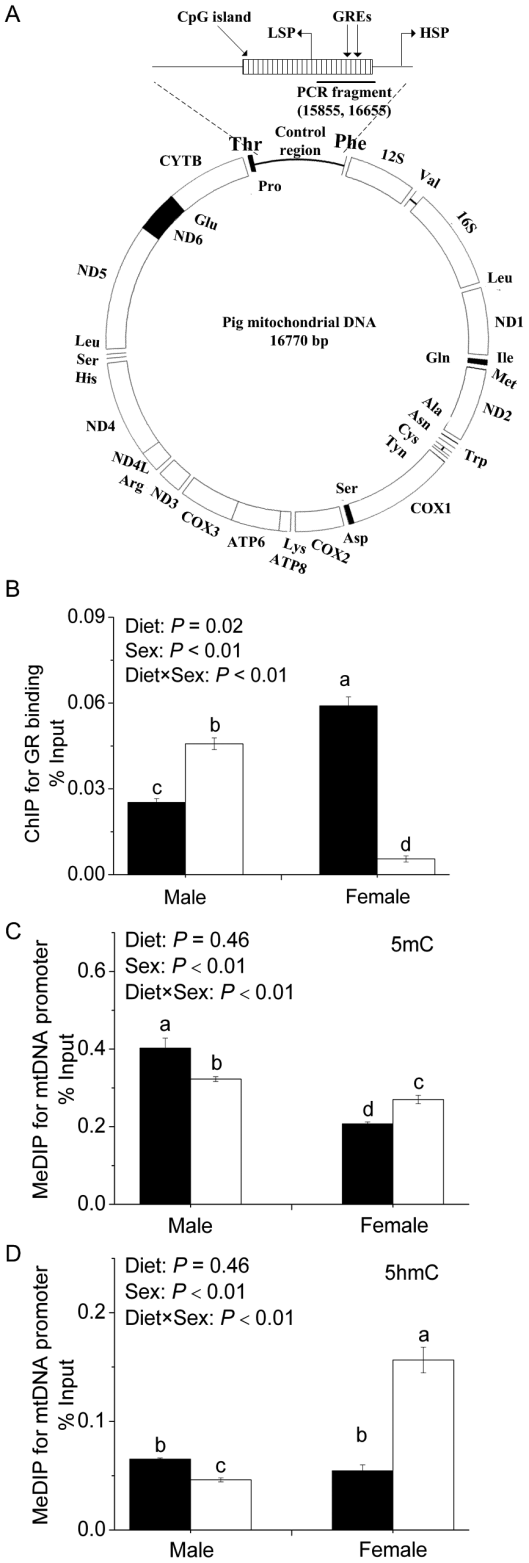
The levels of GR binding and 5(h)mC modifications on mtDNA promoter in the liver of newborn piglets. (A) Schematic structure of pig mtDNA promoter. (B) GR binding to mtDNA promoter. (C) DNA methylation status of the CpG island on mtDNA promoter. (C) DNA hydroxymethylation status of the CpG island on mtDNA promoter. Values are mean ± SEM, Means without a common letter differ, *P*<0.05. Filled bar, standard protein diet; blank bar, low-protein diet. GREs, glucocorticoid response elements; HSP, heavy strand promoter; LSP, light strand promoter.

The control region of the mtDNA spans from 15436 to 16770 bp containing a CpG island (from 15856 to 16655 bp) predicted by Methyl Primer Express version 1.0 (Figure 4A). Two putative GR binding sites, which were located respectively within light strand promoter and heavy strand promoter, were predicted by TRANSFAC®Public 6.0. GR binding to the control region of mtDNA was significantly higher (*P*<0.01) in female piglets compared to male piglets. Moreover, significant interaction between diet and sex was observed (*P*<0.01), which indicates sex-specific responses of GR binding to maternal low protein diet (Figure 4B). LP males responded with significantly higher (*P*<0.01) GR binding, whereas LP females demonstrated significantly reduced (*P*<0.01) GR binding to the control region of mtDNA, compared to their SP counterparts of the same gender, respectively.

### 5-methylcytosine (5mC) and 5-Hydroxymethylcytosine (5hmC) status on the control region of mtDNA

5mC and 5hmC modifications on the mtDNA promoter were affected by both sex and diet. In contrary to GR binding, 5 mC modifications were more enriched in males compared to females in SP group (*P*<0.01). Significant diet and sex interaction (*P*<0.01) indicates sex-specific pattern of cytosine methylation (Figure 4C) and hydroxymethylation (Figure 4D) on the control region of mtDNA in the liver of newborn piglets in response to maternal LP diet. Maternal LP diet significantly decreased cytosine methylation (*P*<0.05) and hydroxymethylation (*P*<0.05) on the control region of mtDNA in male piglets, but the opposite was observed in females. LP females showed significantly enriched cytosine methylation (*P*<0.05) and hydroxymethylation (*P*<0.05) on the control region of mtDNA compared to SP females.

## Discussion

Most prenatal nutritional intervention studies use rats or sheep as model for the fetal programming of human metabolic syndrome. Here we use pig as model based on the higher similarities of pig to human in morphology, physiology, metabolism, and omnivorous habits [26], [27]. In the present study, maternal low protein diet throughout pregnancy resulted in IUGR which is indicated by significantly lower birth weight and liver weight in LP offspring. This finding is consistent with previous reports that maternal protein restriction during pregnancy decreases birth weight and reduces liver, brain, heart and kidney weight of offspring piglets [28]–[30]. Moreover, we found that maternal low protein diet-induced IUGR was associated with decreased mtDNA copy number in the liver of male piglets, but not in females. Decreases in mtDNA copy number have been reported in various tissues of different IUGR animal models, such as skeletal muscle of adult rats [31], adipocytes of adult mice [32], and umbilical blood cells of small for gestational age newborns [33]. However, in these studies either only males were used [31], or the sex disparities were overlooked due to mixed sexes [32], [33]. By contrast, Lattuada et al reported that increased mtDNA copy number is observed in human IUGR placenta [34]. This result indicates different tissues have their own adaptations of maternal stress. To our knowledge, this is the first report regarding the sex-specific effect of maternal LP diet on offspring mtDNA copy number in the liver.

The decrease in hepatic mtDNA copy number indicates disrupted mitochondrial biogenesis, which may cause disturbance of the energy homeostasis in the liver. We detected significantly higher hepatic NADH/NAD ratio in both male and female piglets born to sows fed LP diet. Elevated NADH/NAD ratio favors free radical production [35], inhibits TCA cycle and fatty acid oxidation [36], [37], while increases the generation of lactate for hepatic gluconeogenesis [38]. Interestingly, male and female piglets demonstrated different alterations in hepatic energy metabolism, as indicated by hepatic AMP level and energy charge. Male piglets responded to maternal low protein diet with higher AMP, whereas females in LP group demonstrated lower AMP and higher energy charge. Elevated AMP level causes activation of AMP-activated protein kinase (AMPK) [39], [40], and AMPK activation is associated with increased expression of multiple mitochondrial genes in human skeletal muscle [41]. Therefore, it appears that different mechanisms of energy metabolism are applied in male and female piglets in coping with maternal protein deficiency during prenatal development.

Indeed, five of the thirteen mitochondrial DNA-encoded OXPHOS genes were up-regulated in the liver of male LP piglets, which was accompanied with higher COX enzyme activity. Similar results have been reported previously in rat or mice offspring of malnourished dams [32], [42], [43], but the sex difference was not mentioned. The mechanisms underlying the sex-specific adaptation of mitochondria OXPHOS to maternal malnutrition remain unclear. The structural and functional differences of mitochondria between sexes per se may play a role. Male rats show lower mitochondrial oxidative capacity [44] and higher oxidative stress [45] compared to females, which render males more susceptible to diseases associated with mitochondria dysfunction [46]. Early studies have shown that glucocorticoid stimulates mitochondrial RNA synthesis [47], [48], thereby contributes to the regulation of mtDNA encoded OXPHOS gene expression and protein synthesis in mitochondria. Maternal undernutrition (50% food restriction) during late gestation significantly increases plasma corticosterone in newborn rats [49]. However, we did not detect difference in serum cortisol level between LP and SP newborn piglets. Serum cortisol level in newborn piglets is 3–4 folds higher than the adult level in this study and other publications [50], [51]. Therefore, it is possible that the cortisol surge at birth may have concealed the effect of maternal LP diet. It is noted that male piglets tended to have higher serum cortisol level compared to females, but it is unknown whether the sex difference in cortisol, and/or its interaction with sex steroids, may contribute to sex-specific response in mitochondria OXPHOS gene expression to maternal protein restriction.

Cortisol is a signaling molecular for maternal stress exerting its biological activity through GR. Although we did not detect diet effects on serum cortisol level, GR mRNA abundance was significantly up-regulated in LP piglets, suggesting enhanced cortisol signaling in response to maternal LP diet. GR has been shown to translocate to mitochondria to regulate mtDNA-encoded gene transcription in HepG2 cells [19]. The mitochondrial content of GR protein in the liver of newborn piglets was not affected by maternal dietary protein level, yet GR binding to the control region of mtDNA was significantly altered in a sex-specific manner. To our knowledge, this is the first evidence that maternal LP diet induces a sex-specific GR binding to control region of mtDNA to regulate mtDNA transcription in the liver. A similar sex-specific effect was reported in rat fetuses, where maternal low protein diet during pregnancy up-regulates pancreatic islet ND4L and COX1 expression only in males [11]. It is noted that females demonstrate almost 2-folds higher GR binding to the control region of mtDNA compared to males in SP group, which may contribute to higher oxidation capability reported previously in females [44]. In contrast to males, maternal low protein diet significantly abolished GR binding to the control region of mtDNA, but the mRNA abundances of mtDNA-encoded genes were not affected. We have no interpretation on such seemingly contradictory observation. It was reported that estrogen induces accumulation of the mitochondrial ribonucleic acid in pituitary tumor cells [52], which implies that estrogen may elongate the half-life of mtDNA-encoded mRNAs. In general, the sex disparity is a complex issue, which may be attributable to fetal growth trajectory, and the plasticity in response to the hormonal milieu such as sex hormones, adipokines and cytokines [53]–[55]. Further functional analyses, both in vivo and in vitro, are required to understand the sex-specific mechanisms by which GR binding to mtDNA promoter is regulated in response to maternal low protein diet.

Epigenetic mechanism plays an important role in maternal nutritional programming. Maternal LP diet throughout pregnancy induces hypomethylation of GR and/or peroxisome proliferator-activated receptor alpha gene promoters in male offspring rats at weaning [21] or in adults [56]. Our previous publication also reported that maternal LP diet causes hypomethylation of *G6PC* gene promoter in the liver of male piglets [23]. In this study, we detected reduced 5mC and 5hmC on mtDNA promoter in male piglets born to LP sows, which is associated with enhanced mtDNA transcription. In female offspring, however, both 5mC and 5hmC were more enriched in mtDNA promoter of LP piglets, although no consequences on mtDNA-encoded OXPHOS gene expression were observed. Prenatal exposure to malnutrition generally induces sex-specific modifications in DNA methylation, as reported in human [57] and sheep [58], yet the mechanism underlying this gender disparity is widely unknown. It has been proposed that interactions between sex hormones and DNA methyltransferases may play a role [59]. Although GR binding to mtDNA promoter seems to be inversely correlated with 5mC and 5hmC modifications, it remains to be clarified whether GR binding is affected by DNA methylation and hydroxymethylation, and how epigenetic modifications on mtDNA promoter is regulated, in sex-specific pattern, in the liver of piglets in response to maternal low protein diet.

In conclusion, our results suggest that a maternal LP diet during gestation affects mitochondrial OXPHOS in the liver of newborn piglets in a sex-specific manner through the combined actions of glucocorticoid receptor binding and DNA methylation and hydroxymethylation on the mtDNA promoter. Neonatal changes in hepatic OXPHOS may manifest in adulthood, causing long-term consequences on energy homeostasis. Our findings may provide new insights into the etiology of sex-biased metabolic diseases in human.

## Materials and Methods

### Ethics Statement

The experiment was conducted following the guidelines of Animal Ethics Committee at Nanjing Agricultural University, China. The slaughter and sampling procedures complied with the “Guidelines on Ethical Treatment of Experimental Animals” (2006) No. 398 set by the Ministry of Science and Technology, China and “the Regulation regarding the Management and Treatment of Experimental Animals” (2008) No. 45 set by the Jiangsu Provincial People's Government.

### Animals and experimental design

Fourteen primiparous purebred Meishan gilts obtained from the National Meishan Pig Preservation and Breeding Farm at Jiangsu Polytechnic College of Agriculture and Forestry, Jurong, Jiangsu Province, P. R. China were assigned randomly into standard- (SP) and low- (LP) protein groups. Sows were fed diets containing either 12% crude protein in SP group or 6% crude protein in LP group (Table 3). The dietary treatment started from the first observation of estrus, and artificial insemination was performed at the second estrus. A mixture of semen samples obtained from two littermate boars were used for artificial insemination and the fertilization rate was not influenced by maternal dietary treatment. Sows were fed twice daily (0800 and 1400 h) with rations of 1.8 kg/day during gestation. Newborn piglets were individually weighed immediately after parturition. One male and one female piglet (per experimental group and sex, n = 7) of the mean body weight (±10%) were selected from each litter and killed before sucking. Blood was collected immediately, and the liver (without the gall bladder) samples were snap-frozen in liquid nitrogen immediately and stored at −80°C for further analysis.

**Table 3:** Composition and nutrient content of experimental diets.

	SP	LP
Ingredient (*g/kg*)		
Corn	580	528
Soybean meal	120	0
Bran	150	110
Bone meal	10	5
Corn sugar	100	270
CaHPO_4_	0	7
Fiber^1^	10	10
Attapulgite	0	30
Premix^2^	40	40
Calculated composition		
Digestible energy (*MJ/kg*)	13.1	13.1
Crude protein (%)	12.1	6.1
Crude fiber (%)	2.7	2.3
N-free extracts	640.7	713.0
CP	135.9	67.7
protein∶carbohydrate^3^	1∶10.5	1∶4.7
Calcium (%)	1.2	1.2
Phosphorous (%)	0.4	0.4

1 The fiber concentrate ARBOCEL®was purchased from JRS (Germany)

2 The premix contains (per kilogram): vitamin A: 72 mg; cholecalciferol.: 1.5 mg; vitamin E: 720 mg; menadione: 30 mg; vitamin B-1: 30 mg; vitamin B-2: 120 mg; vitamin B-6: 60 mg; vitamin B-12: 360 mg; niacin: 600 mg; pantothenate: 300 mg; folate: 6 mg; manganese sulfate: 1.0 g; zinc oxide: 2.5 g; iron sulfate: 4 g; copper sulfate: 4 g; sodium selenite: 6 mg; calcium: 150 g; phosphorus: 15 g; sodium chloride: 40 g.

3 Protein-to-carbohydrate ratio based on N-free extracts/CP.

### Radioimmunoassay

Serum concentrations of cortisol (D10PZB), T3 (A01PZB), and T4 (A02PZB) were measured using respective commercial RIA kits (Beijing North Institute of Biological Technology) with assay sensitivities of 2 µg/L, 0.25 µg/L and 5 µg/L, respectively. The intra- and inter-assay variations were 10% and 15%, respectively, for all the three kits.

### Measurement of ATP, ADP, AMP, NAD and NADH levels in liver

Concentrations of AMP, ADP, ATP, NAD, and NADH in liver were determined according to previous publications with some modifications [60], [61]. Tissue extracts were prepared from frozen liver using 0.6 M perchloric acid, and the extracts were centrifuged at 10,000× g for 10 min at 4°C. The supernatants were neutralized with 2 M KOH and centrifuged again. The standards of ATP (FLAAS), ADP (A5285), AMP (01930), NAD (N7004) and NADH (N8129) were purchased from Sigma. High performance liquid chromatography (HPLC) was performed with a reverse-phase column (99603, C18, 5 µm, 250×4.6 mm, Dikma Technologies Inc.) and the column temperature was set at 25°C. For measurements of metabolites, a mobile phase consisting of 215 mM KH_2_PO_4_, 1.2 mM tetrabutylammonium bisulfate, 1% acetonitrile (pH 6.0) was used and the flow rate was maintained at 0.8 mL·min^−1^ by a HPLC pump (600E, Waters). Eluted samples were detected at 260 nm with a dual λ absorbance detector (2478, Waters). Calibration curves were prepared by a six-point standard (0.2, 0.1, 0.05, 0.025, 0.0125 and 0.00625 mg·mL^−1^) of ATP, ADP, AMP NAD and NADH in 0.6 M perchloric acid, respectively.

### Determination of mtDNA copy number

Total genomic DNA was isolated from liver samples and the mtDNA copy number was determined using real-time PCR as previously described with some modifications [62]. Primers specific for the control region of mitochondrial DNA were used for the quantification of the mtDNA molecules, whereas primers specific for the glucose-6-phosphatase nuclear gene were used for standardization (Table 4). Relative mtDNA copy number was calculated with 2^−ΔΔCt^ method [63].

**Table 4:** Primers.

Name	Sequence	Product (bp)
mRNA expression		
* COX1*	F: tggtgcctgagcaggaatagtg	88
	R: atcatcgccaagtagggttccg	
* COX2*	F: gcttccaagacgccacttcac	154
	R: tgggcatccattgtgctagtgt	
* COX3*	F: ggctacagggtttcacgggttg	130
	R: tcagtatcaggctgcggcttca	
* ND3*	F: agcacgcctcccattctcaat	172
	R: tgctaggcttgctgctagtagg	
* CYTB*	F: ctgaggagctacggtcatcaca	162
	R: gctgcgagggcggtaatgat	
* ND1*	F: tcctactggccgtagcattcct	165
	R: ttgaggatgtggctggtcgtag	
* ND2*	F: atcggagggtgaggagggctaa	191
	R: gttgtggttgctgagctgtgga	
* ND4L*	F: gatcgcccttgcagggttactt	182
	R: ctagtgcagcttcgcaggct	
* ND4*	F: tcgcctattcatcagtaagtca	174
	R: ggattatggttcggctgtgta	
* ND5*	F: cggatgagaaggcgtaggaa	103
	R: gcggttgtataggattgcttgt	
* ND6*	F: actgctatggctactgagatgt	124
	R: cttcctcttccttcaacgcata	
* ATP8*	F: tgccacaactagatacatcc	107
	R: gcttgctgggtatgagtag	
* ATP6*	F: actcattcacacccaccacaca	232
	R: cctgctgtaatgttggctgtca	
* PPIA*	F: gactgagtggttggatgg	116
	R: tgatcttcttgctggtctt	
mtDNA copy number, MeDIP and ChIP		
* G6PC*	F: aagccaagcgaaggtgtgagc	165
	R: ggaacgggaaccacttgctgag	
MP	F: acacaccctataacgccttgcc	149
	R: gggtaggtgcctgctttcgtag	

*ATP8*, ATP synthase F0 subunit 8;*ATP6*: ATP synthase F0 subunit 6; *COX1*, cytochrome c oxidase subunit 1; *COX2*, cytochrome c oxidase subunit 2; *COX3*, cytochrome c oxidase subunit 3, *CYTB*, cytochrome b; *G6PC*, glucose-6-phosphatase; MP, mitochondrial DNA promoter; *ND1*, NADH dehydrogenase subunit 1; *ND2*, NADH dehydrogenase subunit 2; *ND3*, NADH dehydrogenase subunit 3; *ND4*, NADH dehydrogenase subunit 4; *ND4L*, NADH dehydrogenase subunit 4 L; *ND5*, NADH dehydrogenase subunit 5; *ND6*, NADH dehydrogenase subunit 6; *PPIA*, peptidylprolyl isomerase A.

### RNA isolation, cDNA synthesis and real-time PCR

Total RNA was isolated from liver samples using TRIzol Reagent (15596026, Invitrogen) according to the manufacturer's instructions and reverse transcribed with the PrimeScript 1st Strand cDNA Synthesis Kit (D6110A, Takara). Two microliters of diluted cDNA (1∶2000) were used in each real-time PCR assay with Mx3000P (Stratagene) and three technical replicates were analyzed for each biological replicate. All primers (Table 4) were synthesized by Generay Biotech (Shanghai, China). Peptidylprolyl isomerase A was chosen as a reference gene and is not affected by the experimental factors (diet & sex).

### COX enzyme activity assay

Liver mitochondria were isolated according to a previously described protocol with some modifications [64]. Two hundred micrograms of frozen liver samples were homogenized in a Dounce homogenizer with 20 strokes using the isolation buffer (w∶v = 1∶5) containing 0.1 M Tris–MOPS, 0.1 M EGTA/Tris, and 1 M sucrose (pH 7.4), and protease inhibitor cocktail (11697498001, Roche) was added to the isolation buffer. The homogenates were centrifuged at 600 *g* for 10 min at 4°C. The supernatant was collected, and then centrifuged at 7000 *g* for 10 min at 4°C. The supernatant was discarded and the pellet was resuspended in the isolation buffer. The suspensions were centrifuged at 7000 *g* for 10 min at 4°C. The final washed mitochondrial pellet was dispersed with the isolation buffer and stored at −70°C until assayed. All operations were carried out on ice.

Mitochondrial protein concentration was measured by a BCA protein assay kit (23225, Pierce). COX activity in liver mitochondria was determined according to the instruction of a commercial kit (GMS10014.2, Genmed Scientifics, Inc.). In brief, 5 µg of the mitochondrial protein were incubated with 100 mM reduced ferrocytochrome *c*, and COX activity was measured at 25°C by the decrease of reduced cytochrome *c* in absorption at 550 nm.

### Mitochondrial 5mC and 5hmC Immunoprecipitation

MeDIP analysis was performed as previously described [23]. Purified total genome DNA was sheared to an average length of 300 bp. Two micrograms of fragmented DNA was heat denatured to produce single-stranded DNA and immunoprecipitation was performed overnight at 4°C with 2 µg IgG (12–371, Millipore), anti-5mC (ab10805, Abcam), or anti-5hmC (39999, Active Motif). Pre-cleared Protein A/G PLUS-Agarose (sc-2003, Santa Cruz) was used to immunoprecipitate the antibody/DNA complexes, and the MeDIP DNA was purified. A small aliquot of MeDIP DNA and control input DNA was used to amplify the control region of mtDNA by real-time PCR with specific primers designed with Primer 5 software (Table 4).

### GR protein content in mitochondria

Equal amount of proteins were subjected to 7.5% and 12% SDS/PAGE gels according to the protein molecular weight and separated by electrophoresis, transferred to 0.45 µm pore-size nitrocellulose filter membranes (66485, Pall) and immunoblotted with anti-GR (sc-1004, Santa Cruz, 1∶200) or anti-COX4 (MB0102, Bioworld, 1∶200). HRP-conjugated anti-rabbit antibody (STAR54, Univ-bio, 1∶8000) and HRP-anti-mouse antibody (STAR86P, Univ-bio, 1∶8000) were used as secondary antibodies. Chemiluminescent substrate (ECL) kit (34080, Pierce) was used to visualize the interested protein bands. The ECL signal intensities were quantified using a VersaDoc MP 4000 system (BioRad).

### GR binding to the control region of mtDNA

ChIP analysis was performed as previously described with some modifications [19], [23]. Briefly, 200 mg of frozen liver samples were ground in liquid nitrogen and washed with phosphate-buffered saline containing protease inhibitor cocktail (11697498001, Roche). After cross-linking in 1% formaldehyde, the reaction was stopped with 2.5 M glycine. The pellets were washed with PBS and lysed with SDS lysis buffer containing protease inhibitors. The lysates were sonicated on ice to yield DNA fragments of 200 to 500 bp in length. After pre-clearance of the resulting chromatin with salmon sperm DNA-protein A/G agarose (50% slurry), the immunoprecipitation was performed with 2 µg of a specific GR antibody (sc-1004x, Santa Cruz) or normal IgG (12–370, Millipore) as control overnight at 4°C. Finally, DNA fragments was released by reverse cross-linking from the immunoprecipitated complex at 65°C for 5 h, and the immunoprecipitated DNA was purified. The resulting DNA was used as template for real-time PCR and specific primers were used to amplify genomic sequences in the control region of mtDNA (Table 4).

### Statistical analysis

All data are presented as the mean ± SEM. Except for gene expression, two-way ANOVA was performed to assess the main effects of diet and sex, as well as the interactions between diet and sex using the general linear model of SPSS 17.0. When significant interactions were observed, the LSD post hoc test was conducted to evaluate differences between the groups. If the data were not normally distributed or the variance was not equal, then log10-transformed data were analyzed. T-test for independent samples was used to analyze the effect of maternal diet on mRNA levels in male or female piglets respectively, because we were not able to include triplicates of all samples from both sexes in the same assays. The 2^−ΔΔCt^ method was applied to analyze real-time PCR data [63] and mRNA or protein expression was shown as the fold change relative to the mean of the male or female SP group. Differences were considered significant at *P*<0.05.
